# Bacterial communities in carnivorous pitcher plants colonize and persist in inquiline mosquitoes

**DOI:** 10.1186/s42523-022-00164-1

**Published:** 2022-02-16

**Authors:** Aldo A. Arellano, Kerri L. Coon

**Affiliations:** 1 Microbiology Doctoral Training Program, University of Wisconsin-Madison, Madison, WI 53706 USA; 2 Department of Bacteriology, University of Wisconsin-Madison, Madison, WI 53706 USA

**Keywords:** Microbiota diversity, Life history, Development, *Wyeomyia smithii*, Mosquito

## Abstract

**Background:**

The leaves of carnivorous pitcher plants harbor diverse communities of inquiline species, including bacteria and larvae of the pitcher plant mosquito (*Wyeomyia smithii*), which aid the plant by processing captured prey. Despite the growing appreciation for this microecosystem as a tractable model in which to study food web dynamics and the moniker of *W. smithii* as a ‘keystone predator’, very little is known about microbiota acquisition and assembly in *W. smithii* mosquitoes or the impacts of *W. smithii*-microbiota interactions on mosquito and/or plant fitness.

**Results:**

In this study, we used high throughput sequencing of bacterial 16S rRNA gene amplicons to characterize and compare microbiota diversity in field- and laboratory-derived *W. smithii* larvae. We then conducted controlled experiments in the laboratory to better understand the factors shaping microbiota acquisition and persistence across the *W. smithii* life cycle. Methods were also developed to produce axenic (microbiota-free) *W. smithii* larvae that can be selectively recolonized with one or more known bacterial species in order to study microbiota function. Our results support a dominant role for the pitcher environment in shaping microbiota diversity in *W. smithii* larvae, while also indicating that pitcher-associated microbiota can persist in and be dispersed by adult *W. smithii* mosquitoes. We also demonstrate the successful generation of axenic *W. smithii* larvae and report variable fitness outcomes in gnotobiotic larvae monocolonized by individual bacterial isolates derived from naturally occurring pitchers in the field.

**Conclusions:**

This study provides the first information on microbiota acquisition and assembly in *W. smithii* mosquitoes. This study also provides the first evidence for successful microbiota manipulation in this species. Altogether, our results highlight the value of such methods for studying host-microbiota interactions and lay the foundation for future studies to understand how *W. smithii*-microbiota interactions shape the structure and stability of this important model ecosystem.

**Supplementary Information:**

The online version contains supplementary material available at 10.1186/s42523-022-00164-1.

## Background

The purple pitcher plant (*Sarracenia purpurea*) is a perennial carnivorous plant occurring natively throughout the southern and northeastern United States and parts of Canada. Each plant produces modified leaves (pitchers) that act as pitfall traps within which captured prey drown in collected rainwater [1]. Captured prey serve as an important source of nitrogen and other nutrients deplete in the bog environments *S. purpurea* plants inhabit. However, *S. purpurea* plants do not endogenously encode several classes of degradative enzymes required for the breakdown of prey [2, 3] and instead must rely on the activity of mutualistic aquatic invertebrates and resident bacterial communities (microbiota) for prey digestion and nutrient assimilation [4–7]. The pitcher plant mosquito (*Wyeomyia smithii*) is one such mutualistic aquatic invertebrate commonly found in *S. purpurea. W. smithii* mosquitoes develop exclusively in pitcher plants, where larvae molt through four consecutive aquatic instars and an aquatic pupal stage before emerging as terrestrial adults [4, 8]. In addition to contributing to the mechanical disruption of captured prey, studies of *W. smithii* in natural settings also implicate larvae as key mediators of assembly of *S. purpurea*-associated communities [9–11]. While recent studies have used surveys at geographical scales to parameterize the contribution of W. *smithii* mosquitoes to ecosystem scale processes [12, 13], the species is rarely maintained in continuous laboratory culture. This has led to a lack of studies seeking to experimentally validate and functionally characterize *W. smithii-*mediated effects in the *S. purpurea* system.

Research into mosquito-microbe interactions has grown recently, owing to data supporting important roles for mosquito microbiota (both the communities of microorganisms present in the mosquito itself and/or the aquatic environments in which mosquitoes develop) in regulating several aspects of mosquito biology, including the ability of certain species to transmit pathogens that cause disease in humans and other vertebrates [14]. This particular feature of mosquito biology is due to most mosquito species being ‘anautogenous’, which means adult females must blood feed on a vertebrate host to produce eggs [8, 15]. Less well-known but of fundamental interest is that some mosquito species have evolved to be ‘autogenous’ and can produce eggs without a blood meal [8, 15]. *Wyeomyia smithii* mosquitoes are facultatively autogenous; adult females in northern populations produce eggs without ever blood feeding, while females in southern populations may produce eggs with or without blood feeding [16]. The genetic underpinnings of the transition from blood feeding to obligate non-biting in northern populations have been studied [17, 18]. Studies in other facultatively autogenous mosquito species also suggest that shifts to autogenous lifestyles may be facilitated by microbial enhancement of nutrient acquisition by larvae, which provides resources for egg production by adult females [19]. However, to date no study has examined microbial diversity in naturally occurring or laboratory-reared populations of *W. smithii* or the impact of *W. smithii*- and pitcher-associated microbiota on *W. smithii* fitness.

Here, we used high throughput sequencing of bacterial 16S rRNA gene amplicons to compare microbiota diversity in field-derived *W. smithii* larvae to laboratory-colonized larvae and characterize microbiota acquisition and assembly across *W. smithii* life history. We then developed methods to generate axenic (microbiota-free) *W. smithii* larvae that can thereafter be selectively recolonized with known microbial taxa and assemblages in the laboratory.

## Methods

### Field collections

Samples of pitcher fluid and resident larvae were sampled exhaustively from 35 mature pitchers located in the Cedarburg Bog Natural Area in Saukville, WI. Fluid from each pitcher was homogenized and drawn out using a sterile syringe affixed with pre-autoclaved nalgene tubing. Tubing and syringes were rinsed with sterile water between pitchers and fluid samples were placed immediately on ice for transport back to the laboratory in Madison, WI. All mosquito larvae were removed from each sample using a sterile thin-stem transfer pipette, rinsed through six iterative washes in sterile DNA-free water (Corning, Corning, NY USA), and stored at − 20 °C prior to DNA isolation. Approximately 5-ml of the remaining fluid in each sample was then centrifuged for 20-min at maximum speed (21,300 × *g*) prior to removal of all supernatant and storage of cell pellets at − 20 °C until DNA isolation.

### Laboratory colony and collections

Laboratory-colonized *W. smithii* were conventionally reared in a reach-in light- and temperature-controlled incubator (Percival) at 25 °C, > 70% relative humidity, and 16-h light: 8-h dark photoperiod [17]. Newly hatched larvae from eggs laid ~ 72-h previously were maintained in covered plastic rearing trays containing distilled water and fed a nutritionally complete, standard diet consisting of guinea pig chow (PMI Nutrition International, Brentwood, MO USA) and freeze-dried brine shrimp (San Francisco Bay Brand, Newark, CA USA) (4:1). Resulting pupae were rinsed in distilled water and resuspended in 50-ml of distilled water before being transferred to cages (BioQuip) for adult emergence. After emergence, conventionally reared adults were provided 5% sucrose in water and rehydrated pesticide-free raisins (Sun-Maid, Fresno, CA USA) ad libitum. Adult females thereafter laid eggs (i.e., oviposited) in containers containing 50-ml distilled water ~ 5 days post-emergence.

Six sets of laboratory-colonized *W. smithii* samples were collected for downstream sequencing: (i) 50-ml of water from four replicate rearing trays containing conventionally reared larvae that had molted to the final (fourth) instar; (ii) four pools of ~ 80 fourth instar larvae from the same rearing trays; four pools of (iii) 10 newly emerged adults (male and female) and (iv) 10 mature adults (male and female), which emerged from surface-sterilized pupae collected from the same rearing trays, (v) egg masses oviposited onto sterile filter paper in sterile water by mature adult females from surface-sterilized pupae (hereafter referred to as ‘STR eggs’), and (vi) egg masses oviposited by conventionally reared females (hereafter referred to as ‘CNV eggs’). Newly emerged adult, mature adult, and STR egg samples were specifically generated as follows. Pupae produced from conventionally reared larvae were surface-sterilized by placing in 2% bleach for 2-min and rinsing 3 × in sterile water. Surface-sterilized pupae were then placed in sterile water in a sterile plastic chamber for adult emergence. Newly emerged adults were collected immediately (< 12-h) after emergence, while mature adults were held in sterile cages and provided sterile 5% sucrose in water for food and sterile filter paper in water for oviposition for 5-days prior to collection of STR egg and mature adult male and female samples.

All water and larval samples were processed immediately after collection as described above. Newly emerged and mature adults were separated by sex using body size, rotation of the male terminalia, and differential terminalia morphology determined using a Leica S9E stereo microscope [20]. Individual adult males and females were then surface-rinsed with 70% EtOH, 0.05% bleach, and sterile water, and finally decapitated to account for eye pigments associated with PCR inhibition [21] prior to pooling and storage at − 20 °C. Egg masses (STR and CNV) were not surface-rinsed and immediately frozen at − 20 °C until DNA isolation.

### Bacterial 16S rRNA library construction and sequencing

Total genomic DNA was isolated from all field and laboratory samples using a standard phenol–chloroform extraction procedure [22, 23] prior to one-step PCR amplification of the V4 region of the bacterial 16S rRNA gene using barcoded primers as described previously [24]. PCR amplification was performed in 25-ul reactions containing ~ 10-ng of template DNA, 12.5-ul of 2X HotStart Ready Mix (KAPA Biosystems, Wilmington, MA USA), and 5-pmol of each primer. No-template reactions as well as reactions using template from blank DNA extractions served as negative controls. Reaction conditions were: initial denaturation at 95 °C for 3-min, followed by 25 cycles at 95 °C for 30-s, 58 °C for 30-s, and 72 °C for 30-s, and a final extension step at 72 °C for 5-min, with the exception of all adult samples derived from our standard *W. smithii* laboratory colony, which were amplified at 30 cycles. Products were visualized on 1% agarose gels and purified using a MagJET NGS Cleanup and Size Selection Kit (Thermo Fisher Scientific) or by running the entire reaction volume on a 1% low-melt agarose gel prior to DNA recovery from bands of the correct size using a ZR-96 Zymoclean Gel DNA Recovery Kit (Zymo Research, Irvine, CA USA). The 91 resulting purified libraries were finally quantified using a Quantus fluorometer (Promega) and combined in equimolar amounts prior to paired-end sequencing (2 × 250-bp) on an Illumina MiSeq by the DNA Sequencing Facility at the University of Wisconsin-Madison (Madison, WI USA).

### Sequencing data analyses

De-multiplexed reads were imported into QIIME2 [25] and paired reads joined using VSEARCH [26]. Denoising was then carried-out in two steps; first, reads were quality-filtered using q-scores [27], and second, Deblur was used with `p-trim-length` set to the position at which point the median quality score prior to filtering begins to drop, here corresponding to a value of 250 [28]. Taxonomy was assigned using a Naive-Bayes classifier trained using Greengenes reference sequences [29, 30]. Multiple sequence alignment was performed using `mafft` [31], and phylogenetic tree construction was performed using FastTree2 [32]. All endpoint artifacts generated in QIIME2 were then exported, merged with metadata, and converted to a phyloseq object for further analysis in R (version 4.1.1) [33].

Rooting of the phylogenetic tree was performed in R using phyloseq and a decontamination procedure was implemented using a two-tiered approach implemented in the R package ‘decontam’ [34]. DNA quantification values prior to library pooling in study samples, blank DNA extraction products, and PCR negative controls were used to generate a list of likely contaminant reads. Contaminant reads that were more prevalent in control samples than in study samples were then removed from the entire dataset, along with samples with fewer than 100 total reads and reads classified as ‘Chloroplast’ or ‘mitochondria’ prior to downstream analyses.

Species richness and Shannon diversity were estimated using the R packages ‘breakaway’ and ‘DivNet’, respectively [35, 36]. DivNet offers the functionality of covariate-wise alpha diversity index estimation in addition to sample-wise estimation—here both metrics are reported with heavily outlying samples removed prior to covariate-wise Shannon diversity estimation. Statistical tests of covariate-wise differences in estimated richness or Shannon diversity were computed using the ‘betta’ function in the R package ‘breakaway’ [37]. Beta diversity ordinations were constructed using both phylogeny-aware and phylogeny-unaware metrics appropriate for compositional data analysis (CoDa) [38]. In the former case, we implemented PhILR transformation (Phylogenetic Isometric Log-Ratio), which uses the phyloseq abundance table and phylogenetic tree to generate values termed ‘balances’ that represent the log-ratio of the geometric mean abundance of taxa that descend from a given internal node on the provided phylogenetic tree [39, 40]. In the latter case, a centered-log ratio (clr) transformation was used in the R package ‘microbiome’ [41]. For both metrics, transformation was followed by principal component analysis (PCA) and beta diversity ordination using phyloseq against the two most explanatory principal component axes. Global statistical differences in clustering by covariates of interest were determined using PERMANOVA (permutational multivariate analysis of variance) implemented using the function ‘adonis’ in the R package ‘vegan’ [42]. Subsequent pairwise tests were undertaken in the event of a significant global test and p-values corrected using the Bonferroni method. The instance of variance-driven significant results was assessed using PERMDISP (permutational analysis of multivariate dispersions) using the ‘betadisper’ function in the R package ‘vegan’ with pairwise tests also conducted and corrected via the Bonferroni method when the global test was significant [42]. Differences in taxon abundance across sample groups were tested for significance using ALDEx2 and Benjamini-Hochberg (FDR) adjusted *p*-values [43].

### Preparation of axenic and gnotobiotic larvae

Axenic larvae were produced by placing eggs derived from the conventional laboratory colony into sterile Petri dishes containing 70% EtOH for 5-min, transferring to a solution of 5% bleach and 0.01% D-256 disinfectant (Vedco, Saint Joseph, MO USA) for 3-min, transferring again to 70% EtOH for 5-min, and rinsing 3 × in sterile water. Eggs were then transferred to vented 25-cm^2^ cell culture flasks (Corning) containing 30-ml of sterile water and 2.5 ug/ml of amphotericin B (Fisher BioReagents) and incubated under conventional rearing conditions (described above). First instars hatched 48–72-h later and were fed standard diet (described above) sterilized by exposure to 10 kGy from a cobalt 60 gamma radiation source housed in the Breazeale Nuclear Reactor Building on The Pennsylvania State University campus (University Park, PA USA). Sterility of larvae and diet were confirmed by culture-based and PCR analysis using universal bacterial 16S rRNA gene and fungal ITS primers as described previously [44].

Gnotobiotic (i.e., recolonized) larvae were produced by inoculating a given microbiota treatment into replicate wells of sterile 6-well culture plates (Corning) containing 5-ml of sterile water, 10 axenic first instars, and 3-mg of sterilized diet. Plates were then maintained under conventional rearing conditions (described above) and fed and monitored daily for larval growth and molting. One of three microbiota treatments (i.e., inocula) were used to produce gnotobiotic larvae in this study: (i) 5-ul of material from a cryopreserved glycerol stock containing the mixed community of bacteria present in the water of fourth instar larvae under conventional rearing conditions (~ 5 × 10^5^ total bacterial cells), (ii) ~ 5 × 10^5^ cells of one of four microbial taxa isolated from fluid collected from pitchers in the field (described above), or (iii) ~ 5 × 10^5^ cells of a standard laboratory strain of *Escherichia coli* (str. K12 substr. MG1655). Maintenance of gnotobiotic conditions was confirmed in a subset of samples by streak-plating on permissive media and confirming growth by the single morphotype of interest.

### Growth measurements and data analysis

Growth of gnotobiotic larvae in response to different microbiota treatments was measured as the proportion of larvae that developed to the pupal stage and the total development time (days) of individual larvae to pupation. Pupae from plates inoculated with the mixed community of bacteria present under conventional rearing conditions were also pooled by sex as determined by genital lobe morphology using a Leica S9E stereo microscope, surface-sterilized as described above, and allowed to emerge from sterile water in sterile chambers to measure the impact of microbiota recolonization on adult body size (measured as the length of the right forewing from the axillary incision to the tip excluding fringe) [45, 46]. All wing length measurements were conducted using a Leica S9i digital stereo microscope, LAS EZ image capture software, and ImageJ. All microbiota treatments were assayed using two independent cohorts of axenic larvae, resulting in at least two plates, sixteen wells, and ~ 120 larvae being assayed per microbiota treatment. Larvae reared in the absence of any microbes and maintained alongside experimental plates served as the negative control for all experiments.

Proportional data (e.g., survival to pupation) were analyzed by Bonferroni-corrected pairwise Barnards’s or Chi-square tests to compare axenic and gnotobiotic treatments to the conventional positive control. Non-proportional data (e.g., days to pupation) confirmed to meet the assumptions of parametric statistical tests were analyzed using Student’s *t*-tests (two groups of equal variance), Welch’s *t*-tests (two groups of unequal variance), or one-way analysis of variance (ANOVA) followed by post hoc Tukey–Kramer Honest Significant Difference (HSD) tests for multiple comparisons with Bonferroni correction. Non-parametric data were analyzed using Mann Whitney U tests (two groups) or Kruskal–Wallis tests (three or more groups) followed by post hoc Dunn’s tests for multiple comparisons with Benjamini-Hochberg (FDR) adjusted *p*-values.

## Results

### Laboratory colonization reduces diversity and shifts composition of the *W. smithii* larval microbiota

We first characterized the bacterial community present in laboratory colonized *W. smithii* larvae and compared it to the community present under field conditions. Sequencing of 16S rRNA gene amplicons from water and larval samples derived from either naturally occurring pitchers in the Cedarburg Bog State Natural Area in Wisconsin, USA or four replicate rearing trays from our standard rearing colony in the laboratory generated a total of 786,262 sequences (median = 9701 per sample) that were assigned to 761 unique ASVs after quality control filtering (Additional file 1: Table S 1). Rarefaction curves saturated at 1000 sequences for all but one sample, which was eliminated from the dataset prior to downstream analyses (Additional file 1: Table S 1, Additional file 3: Fig. S 1). Sample complexity varied with total ASVs ranging from 25 to 242 for all water and 21 to 122 for all larval samples (Additional file 1: Table S 1). Bacterial diversity was higher in field-derived samples than laboratory-derived samples as measured by both breakaway richness (*p* < 0.001) and the Shannon index (*p* < 0.001) (Additional file 1: Table S 1). Bacterial diversity was also higher in water than larvae, though to a greater extent in the field than in the lab (field: breakaway richness *p* < 0.001, Shannon diversity *p* < 0.001); lab: breakaway richness *p* = 0.64, Shannon diversity *p* = 0.002) (Additional file 1: Table S 1).

**Table 1:** PERMANOVA and PERMDISP analysis of the effect of sample source (field vs. lab) and sample type (larvae vs. water) on beta diversity of *W. smithii*-associated bacterial communities as measured by PhILR

Comparison	PERMANOVA		PERMDISP	
	Test statistic	*p*-value	Test statistic	*p*-value
Overall (F)	F_3,58_ = 11.82	0.001**	F_3,58_ = 12.96	0.001**
Field larvae–field water	19.62	0.006**	− 1.82	0.32
Field larvae–lab larvae	24.21	0.006**	4.23	0.006**
Field larvae–lab water	30.51	0.006**	2.85	0.04*
Field water–lab larvae	14.18	0.006**	5.80	0.006**
Field water–lab water	18.81	0.006**	4.09	0.006**
Lab larvae–lab water	1.32	1	− 0.99	1

* *p* < 0.05, ** *p* < 0.01

**Fig. 1:**
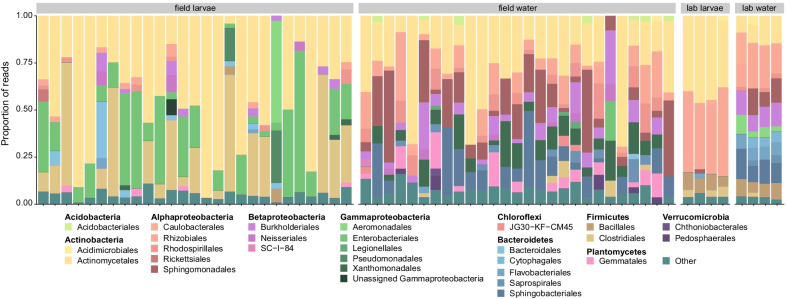
Relative abundance of bacterial orders in water and *W. smithii* larvae from naturally occurring pitchers in the field and the laboratory. Each bar presents the proportion of sequencing reads assigned to a given order. Only categories > 2% are presented

**Fig. 2:**
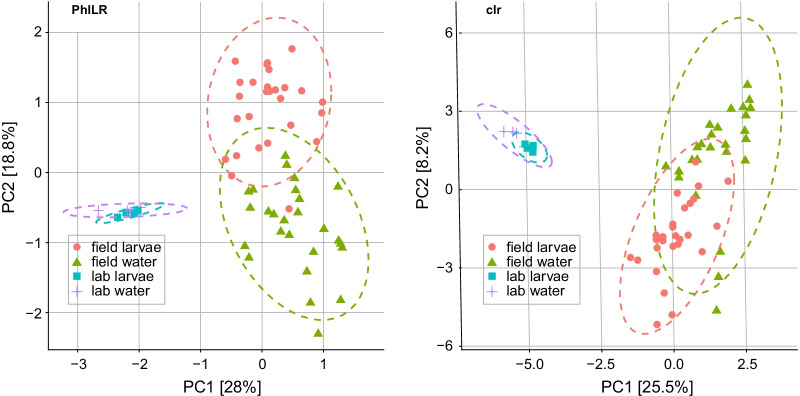
Ordination analyses using phylogenetic aware (PhILR, *left*) and unaware (clr, *right*) beta diversity indices. Legends in the bottom left of each plot designate sample type by the following symbol shapes/colors: red circles (field-derived larvae), green triangles (field-derived water), blue squares (lab-derived larvae), and purple crosses (lab-derived water). Ellipses designate 95% confidence intervals. Permutational multivariate analysis of variance (PERMANOVA) and permutational analysis of multivariate dispersions (PERMDISP) were used to test for group effects and heterogeneity of dispersion, respectively (see Tables 1, 2)

Fifteen bacterial phyla were identified across all samples, but four accounted for ~ 95% of ASVs: Proteobacteria (44%), Actinobacteria (32%), Bacteroidetes (11%), and Firmicutes (8%). Classification into orders showed that larvae contained the same taxa present in the water they were collected from, but relative abundance differed (Fig. 1; Additional file 4: Fig. S 2). Laboratory-colonized larvae also harbored bacterial communities that significantly differed in composition from field-collected larvae. Laboratory-colonized *W. smithii* larvae contained a notably greater proportion of taxa within the orders Sphingobacteriales (*p* = 0.01), Aeromonadales (*p* = 0.03), Flavobacteriales (*p* = 0.02), Cytophagales (*p* = 0.001), Bacillales (*p* = 0.005), and Rhizobiales (*p* = 0.001) than field-collected larvae, while field-collected larvae contained a greater proportion of taxa within the orders Enterobacteriales (*p* = 0.001), Xanothomonadales (*p* = 0.007), and Gemmatales (*p* = 0.02) (Fig. 1). These observations were further supported by ordination analyses using both phylogenetic aware (PhILR) and unaware (clr) beta diversity indices, both of which revealed significant clustering of samples by collection site (field vs. lab) with bacterial communities in larvae being most similar to those in the water from which they were collected (Fig. 2; Tables 1, 2). Only twelve ASVs were shared across all of the field and laboratory larval samples we sequenced. These ASVs belonged to one of seven bacterial orders (Actinomycetales, Acidimicrobiales, Rhizobiales, Sphingomonadales, Aeromonadales, and Clostridiales) and included members of genera *Cryocola*, *Sphingomonas*, *Bradyrhizobium*, *Elizabethkingia*, and *Clostridium*. Additionally, common ASVs mapped to unidentified members of the Lachnospiraceae, Microbacteriaceae, Acidimicrobiales, Aeromonadaceae, and Rhizobiales. Bacterial taxa detected in field-collected larvae that were absent in laboratory-colonized larvae included 35 ASVs distributed across 15 bacterial orders and 27 genera, including members of the Xanthomonadaceae and Neisseriaceae, known to commonly associate with *S. purpurea* in bog environments [4, 5, 47–49]. In contrast, taxa unique to laboratory-colonized larvae included only two ASVs from the order Pseudomonadales and of the genera *Pseudomonas* and *Acinetobacter*, both of which are common in laboratory colonies of other mosquito species [50–57].

**Table 2:** PERMANOVA and PERMDISP analysis of the effect of sample source (field vs. lab) and sample type (larvae vs. water) on beta diversity of *W. smithii*-associated bacterial communities as measured by clr

Comparison	PERMANOVA		PERMDISP	
	Test statistic	*p*-value	Test statistic	*p*-value
Overall (F)	F_3,58_ = 8.10	0.001**	F_3,58_ = 9.82	0.001**
Field larvae–field water	11.87	0.006**	− 3.21	0.03**
Field larvae–lab larvae	31.31	0.006**	3.05	0.02*
Field larvae–lab water	40.96	0.006**	2.38	0.04*
Field water–lab larvae	16.40	0.006**	3.56	0.006**
Field water–lab water	20.18	0.006**	3.11	0.02**
Lab larvae–lab water	2.31	0.18	− 0.55	1

* *p* < 0.05, ** *p* < 0.01

### Sequence-based profiling of bacterial diversity across *W. smithii* life history

Next, we used 16S rRNA gene amplicon sequencing to assess whether bacteria are transstadially transmitted from *W. smithii* larvae to adults by surface-sterilizing pupae from our standard laboratory rearing colony and allowing adults to emerge from sterile water in a sterile chamber. A subset of newly emerged adults (male and female) was then processed shortly (< 12-h) after emergence, while the remaining adults were held in sterile chambers containing only a sterile sucrose solution for consumption in order to assess bacterial persistence to maturity. Mature adult females were finally allowed to oviposit eggs under sterile conditions to assess the ability of *W. smithii* mosquitoes to disperse bacteria into the pitcher environment and between mosquito generations. The resulting sequencing dataset contained a total of 236,271 sequences (median = 7294 per sample) assigned to 191 unique ASVs across a total of 26 samples (Additional file 2: Table S 2), including the water and larval samples included in our comparative analyses with field-collected material (described above). Rarefaction curves saturated at 1000 sequences for all but the newly emerged adult samples (Additional file 2: Table S 2, Additional file 5: Fig. S 3), for which we generated significantly fewer reads on average than for other samples (ANOVA on log-transformed total reads: F_(3,18)_ = 32.6, *p* < 0.001; followed by a Tukey–Kramer HSD test). Two of these samples were also identified as ‘heavily outlying’ during initial analysis and were therefore removed from the dataset prior to all subsequent analyses (Additional file 2: Table S 2).

**Fig. 3:**
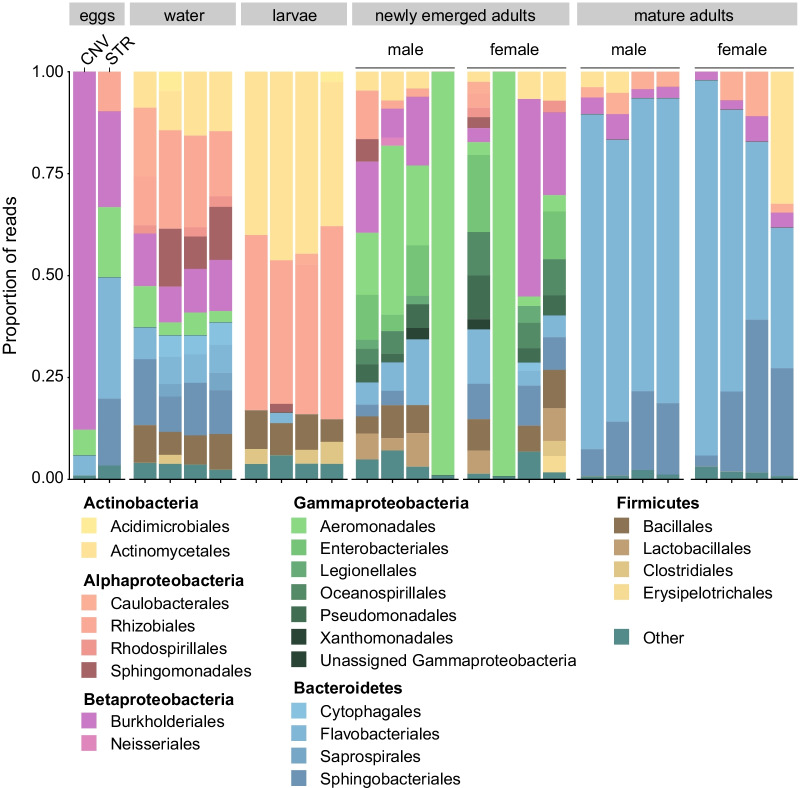
Relative abundance of bacterial orders across *W. smithii* life history in the laboratory. Each bar presents the proportion of sequencing reads assigned to a given order. Only categories > 2% are presented

The final dataset collectively indicated that bacterial diversity declined from 120 ASVs in larvae and larval water to 112 in newly emerged adults and 28 in mature adults (male and female) (Fig. 3; Additional file 2: Table S 2). Indeed, bacterial diversity as measured by the Shannon index was distinct in larvae relative to either newly emerged (*p* < 0.001) or mature adults (*p* < 0.001) (Additional file 2: Table S 2). The majority (> 95%) of bacterial genera detected in mature adults were also present across mosquito development, although there were dramatic shifts in the relative abundance of specific community members (Fig. 3). More specifically, taxa within the most abundant bacterial orders in larvae (Actinomycetales, Rhizobiales) decreased in abundance in newly emerged adults, while taxa within the bacterial orders Burkholderiales, Aeromonadales, and Flavobacteriales increased (Fig. 3). Sugar feeding further reduced the abundance of larval taxa in mature adults, while greatly increasing the abundance of taxa within the bacterial orders Flavobacteriales and Sphingobacteriales (Fig. 3). Shannon diversity was also significantly lower in mature adults than in newly emerged adults (*p* < 0.001), irrespective of sex (male vs. female) (Additional file 2: Table S 2). We did not detect any notable differences in bacterial diversity between male and female newly emerged (*p* > 0.05 for both breakaway richness and Shannon index) or mature (*p* > 0.05 for both breakaway richness and Shannon index) adults (Additional file 2: Table S 2).

**Fig. 4:**
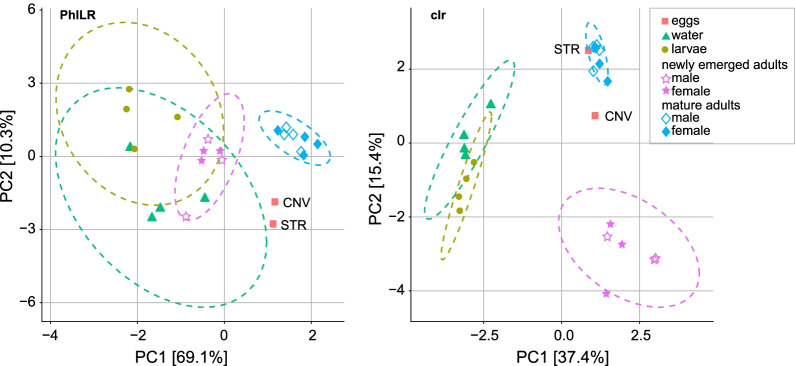
Ordination analyses using phylogenetic aware (PhILR, *left*) and unaware (clr, *right*) beta diversity indices. Legends in the bottom left of each plot designate sample type by the following symbol shapes/colors: red squares (eggs), green triangles (water), yellow circles (larvae), unfilled purple stars (newly emerged adult males), filled purple stars (newly emerged adult females), unfilled blue diamonds (mature adult males), and filled blue diamonds (mature adult females). Ellipses designate 95% confidence intervals. Permutational multivariate analysis of variance (PERMANOVA) and permutational analysis of multivariate dispersions (PERMDISP) were used to test for group effects and heterogeneity of dispersion, respectively (see Tables 3, 4)

While we did not sequence enough samples to robustly compare bacterial diversity on eggs against all of the other samples we sequenced, our results identified two notable patterns worthy of future investigation. First, we detected many (~ 92%) of the ASVs present in mature adult females on STR eggs laid under sterile conditions, including taxa within each of the dominant bacterial orders (e.g., Flavobacteriales, Sphingobacteriales) detected in females prior to oviposition (Fig. 3). Many of these taxa (~ 73%) were also present on both CNV eggs laid by conventionally reared females under non-sterile conditions and all (100%) were detected in the water samples we collected from conventional rearing trays. Second, ordination analyses using both the PhILR and clr beta diversity indices further supported the observation that bacterial communities present on eggs were most similar to those present in mature adult females and the water samples we sequenced (Fig. 4; Tables 3, 4).

**Table 3:** PERMANOVA and PERMDISP analysis of the effect of sample type (i.e., life stage) on beta diversity of *W. smithii*-associated bacterial communities as measured by PhILR

Comparison	PERMANOVA		PERMDISP	
	Test statistic	*p*-value	Test statistic	*p*-value
Overall (F)	F_3,18_ = 24.91	0.001**	F_3,18_ = 1.84	0.17
Larvae–water	1.99	0.82	–	–
Larvae–newly emerged adults	20.54	0.04*	–	–
Larvae–mature adults	115.87	0.02*	–	–
Newly emerged adults–water	12.99	0.05	–	–
Newly emerged adults–mature adults	77.97	0.01*	–	–
Mature adults–water	73.81	0.01*	–	–

* *p* < 0.05, ** *p* < 0.01

**Table 4:** PERMANOVA and PERMDISP analysis of the effect of sample type (i.e., life stage) on beta diversity of *W. smithii*-associated bacterial communities as measured by clr

Comparison	PERMANOVA		PERMDISP	
	Test statistic	*p*-value	Test statistic	*p*-value
Overall (F)	F_3,18_ = 7.64	0.001**	F_3,18_ = 41.23	0.001**
Larvae–water	1.37	1	− 0.70	1
Larvae–newly emerged adults	12.16	0.02*	− 6.19	0.02*
Larvae–mature adults	78.77	0.02*	5.32	0.02*
Newly emerged adults–water	10.68	0.02*	3.29	0.11
Newly emerged adults–mature adults	32.08	0.006**	− 13.38	0.006**
Mature adults–water	57.54	0.02*	− 5.00	0.02*

* *p* < 0.05, ** *p* < 0.01

### Laboratory-colonized *W. smithii* larvae as a tractable model in which to study microbiota function

The final goal of this study was to develop methods to generate axenic *W. smithii* larvae that can thereafter be selectively recolonized with known microbial taxa and assemblages in the laboratory. We first produced axenic larvae by surface-sterilizing eggs and hatching first instars in sterile water. We then plated pooled homogenates of first instars hatched from sterilized eggs and subsequently maintained in plates containing sterile water and diet to confirm that no bacteria or fungi could be cultured on nutrient or blood agar plates. Total genomic DNA was also isolated from pools of axenic larvae to confirm that no amplicons could be generated via PCR with universal bacterial (16 rRNA gene) or fungal (ITS) primers (Additional file 6: Fig. S 4).

Next, we developed methods to generate gnotobiotic *W. smithii* larvae by reintroducing the mixed community of bacteria present under conventional rearing conditions (hereafter referred to as ‘native microbiota’) into the water of cultures containing axenic larvae. We then used these methods to query the larval fitness impacts of individual bacterial isolates derived from naturally occurring pitchers in the field (described above) by comparing the developmental outcomes of gnotobiotic larvae monocolonized by individual isolates to larvae recolonized by their native microbiota. Bacterial isolates were obtained by plating pitcher fluid on various media and then sequencing 16S rRNA gene amplicons from individual colonies. This resulted in isolation of a number of taxa in bacterial orders previously identified by sequencing of the same communities (Fig. 1). We focused our recolonization assays on isolated strains of *Chromobacterium* (Order Neisseriales), *Elizabethkingia* (Order Flavobacteriales), *Paraburkholderia* (Order Burkholderiales), and *Rhizobium* (Order Rhizobiales). These genera were commonly isolated from pitcher fluid and mosquito-derived field samples in addition to being prominent genera in field 16S rRNA amplicon sequencing data. We also conducted recolonization assays using the K12 strain of *E. coli*, as this bacterium was not detected in any of the field or laboratory samples we sequenced.

**Fig. 5:**
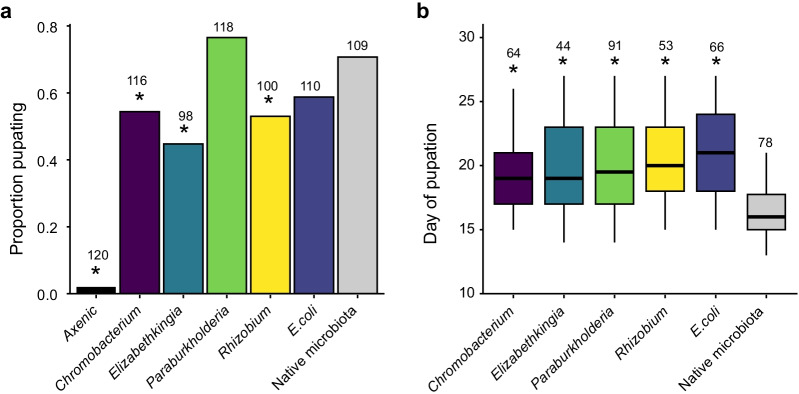
**a** Proportion of axenic first instars surviving to the pupal stage when fed: sterilized diet only (Axenic) or sterilized diet plus different bacterial isolates. Gnotobiotic larvae recolonized with the mixed community of bacteria present under conventional rearing conditions (i.e., their ‘Native microbiota’) served as the positive control. A minimum of 98 larvae were assayed per treatment across two independent assays. An asterisk (*) indicates a significant difference for a given treatment relative to the positive control (Barnard’s test; *p* < 0.01). **b** Development time of the same larvae from egg hatching to pupation. Box-and-whisker plots show high, low, and median values, with lower and upper edges of each box denoting first and third quartiles, respectively. An asterisk (*) indicates a significant difference for a given treatment relative to the positive control (Mann–Whitney U test; *p* < 0.01). The number above each bar represents the number of larvae that pupated and for which development time to pupation was recorded

Results showed that ~ 79% of gnotobiotic *W. smithii* larvae recolonized by their native microbiota developed to the pupal stage on average ~ 17.5 days post-egg-hatching and emerged into adults that were comparable in size to conventionally reared adults from our standard laboratory colony (Fig. 5; Additional file 7: Fig. S  5). Results further showed that each of our bacterial isolates of interest was able to colonize and persist in larvae as evidenced by the ability to recover and culture viable colonies from water and homogenates of fourth instar larvae collected from experimental plates. However, only gnotobiotic larvae monocolonized by *Paraburkholderia* or *E. coli* exhibited pupation rates that did not differ from gnotobiotic larvae recolonized by their native microbiota and all of the bacterial isolates we assayed produced gnotobiotic larvae that developed slower than gnotobiotic larvae recolonized by their native microbiota (Fig. 5). Interestingly, the reduced pupation rate observed for gnotobiotic larvae monocolonized by *Chromobacterium, Elizabethkingia,* or *Rhizobium* was due to a larger proportion of larvae failing to pupate (*χ*^*2*^ = 14.865, *df* = 1, *p* < 0.001) rather than a larger proportion of individuals dying as larvae (*χ*^*2*^ = 0.145, *df* = 1, *p* > 0.05). Axenic *W. smithii* larvae, which served as a negative control for all of our recolonization assays and were provided sterilized diet only throughout the experiment, also failed to grow beyond the first instar with all larvae eventually dying without pupating before the end of the experiment (Fig. 5).

## Discussion

The leaves of carnivorous *Sarracenia* pitcher plants and the aquatic food webs within them have long been considered a model system in ecological research [58–61]. A key constituent of these food webs is the pitcher-associated microbiota, which includes diverse communities of bacteria that are distinct from the surrounding environment and that are essential for digestion of captured prey and nutrient assimilation by the plant [4–7]. The degree to which bacteria can digest prey is thought to be dependent, at least in part, on the abundance of larvae of the endemic mosquito *W. smithii*, which feed on bacteria, protozoans, macroinvertebrates, detritus, and other organic matter present in pitcher fluid [58, 62]. However, while *W. smithii-*induced shifts in the taxonomic and/or functional profiles of pitcher-associated microbial communities have the potential to alter nutrient cycling and other processes to the benefit or detriment of the host plant [9], almost nothing is known about *W. smithii-*microbiota interactions, likely owing to their lack of immediate biomedical relevance and unique biology, which makes them difficult to rear continuously in the laboratory.

The first goal of this study was to characterize microbiota diversity in field-derived *W. smithii* larvae and compare it to that in laboratory-colonized larvae. We then used this information to further characterize microbiota acquisition and assembly across *W. smithii* life history. All mosquito larvae are aquatic and develop by feeding on detritus and other organic matter, including bacteria, present in the surrounding water column [8]. Previous studies in both laboratory and field-collected mosquitoes collectively indicate that mosquito larvae hatch from eggs with no extracellular microbes in their digestive tract [44]. Thereafter, they ingest bacteria and other microorganisms from their aquatic environment, which colonize the gut to form a microbiota that is in part transstadially transmitted to the adult stage [44, 53, 56, 63, 64]. The adult gut microbiota may further change in response to consumption of water from breeding sites, nectar or other food sources including a blood meal, although bacterial diversity in adults is consistently much lower than in larvae [44, 63, 65–69]. Limited evidence also suggests that a portion of the microbiota present in adult females is deposited onto eggs during oviposition, which may provide a weak mode of vertical transmission between generations [44].

Our results identified members of the bacterial phyla Proteobacteria (Neisseriales, Burkholderiales, Enterobacteriales, Xanthomonadales, Pseudomonaldales), Actinobacteria, Bacteroidetes (Sphingobacteriales, Flavobacteriales), and Firmicutes (Clostridiales) as dominant taxa in the fluid of naturally occurring pitchers in the field, consistent with previous studies in *S. purpurea* [4, 5, 47–49]. Members of the same taxa were also detected in *W. smithii* larvae collected from the same pitchers, which supports previous results showing that mosquito larvae are colonized by a subset of the bacteria they ingest during feeding [44, 53, 56, 63, 64]. Further consistent with previous studies was the overall observation that microbiota diversity was much lower in laboratory-reared mosquitoes as compared to field-collected mosquitoes and that field and laboratory populations of mosquitoes exhibited differences in community composition [50–57], including the dominance of taxa within the bacterial orders Sphingobacteriales, Aeromonadales, Flavobacteriales, Cytophagales, Bacillales, and Rhizobiales in laboratory-reared *W. smithii* larvae that were less abundant in field-collected individuals. That we detected a subset of the ASVs present in laboratory-reared *W. smithii* larvae in newly emerged adults from surface-sterilized pupae provides experimental evidence to support largely circumstantial data suggesting adult mosquitoes initially acquire their gut microbiota from larval breeding sites [67]. The reduction of ASV diversity in newly emerged adults and proliferation of specific taxa in mature adults after sugar feeding is also consistent with patterns seen in other studies [65, 70], including the proliferation of taxa within the bacterial orders Sphingobacteriales and Flavobacteriales that overwhelmingly dominated our mature adult samples.

Overall, our sequencing results support a dominant role for the pitcher environment in shaping the microbiota present in *W. smithii* mosquitoes. That we detected dominant taxa present in mature adult females on both (*i*) the surface egg masses, and (*ii*) water from conventional rearing trays in the laboratory also supports a potential role for oviposition by adult female *W. smithii* mosquitoes in dispersing bacteria into the pitcher environment and between mosquito generations. Whether the same patterns are observed under field conditions warrants future study. Whether these patterns are consistent across individual females also warrants future study, given the high degree of inter-individual variation in microbiota diversity observed in other mosquito species [44, 50, 51, 63–65, 69, 71–74].

The second goal of our study was to develop methods to generate axenic *W. smithii* larvae that can thereafter be selectively recolonized with known microbial taxa and assemblages (i.e., to produce gnotobiotic larvae) in the laboratory. We then used these methods to demonstrate our ability to use axenic and gnotobiotic larvae to examine the impact of *W. smithii*- and pitcher-associated microbiota on the growth and development of *W. smithii* larvae to the adult stage. We decided to focus on larval development as our fitness outcome of interest for several reasons. First, previous research in anautogenous (blood-feeding) and autogenous (non-blood-feeding) species spanning the phylogenetic breadth of the mosquito family Culicidae suggests that most (if not all) mosquitoes require a living gut microbiota to develop from larvae into adults under natural conditions [19, 44, 53, 75, 76]. Research in *Aedes aegypti* further indicates that gut microbes regulate mosquito development by inducing gut hypoxia, stabilization of hypoxia-inducible transcription factors (HIFs), and activation of signaling pathways with roles in larval growth and molting [77, 78]. It was therefore of fundamental interest to understand whether the symbiotic association of *W. smithii* with *S. purpurea* has relaxed the requirement for a living gut microbiota for development as previously observed in other mosquito species. Second, adult body size in mosquitoes and other insects is largely determined by larval nutrition and developmental conditions, and adult females of autogenous mosquito species must fully rely on nutrient reserves acquired from feeding during the larval stage to produce eggs [79–82]. In this way, impacts of microbiota composition on larval fitness traits in mosquitoes has important implications for adult fitness traits such as individual body size, teneral reserves, fecundity, and longevity [19, 83]. Finally, *W. smithii* mosquitoes interact most frequently (in space and time) with *S. purpurea* during the larval stage. Thus, whether specific microbiota taxa enhance or reduce *W. smithii* larval fitness is of general interest to understanding the evolution and maintenance of this and other symbioses.

Our results show that axenic *W. smithii* larvae fed sterilized diet under a standard photoperiod and sterile conditions fail to grow beyond the first instar but develop normally when inoculated with the mixed community of bacteria present under conventional rearing conditions (i.e., their native microbiota). Our results also experimentally demonstrate that individual bacterial isolates derived from naturally occurring pitchers in the field, or the standard laboratory model bacterium *E. coli*, can successfully colonize *W. smithii* larvae and persist to the fourth instar. However, only two of the isolates we assayed supported survival rates of gnotobiotic larvae to the pupal stage that did not differ from gnotobiotic larvae recolonized by their native microbiota, and none of the isolates we assayed supported normal development as measured by both pupation rate and the development time of gnotobiotic larvae to pupation. While we did not measure gut hypoxia, HIF stabilization, larval growth and/or activation of specific signaling pathways previously demonstrated to be regulated by microbiota colonization in other mosquito species, these results strongly support a conserved role for a living gut microbiota in regulating the development of *W. smithii* mosquitoes. This species appears to rely on environmentally acquired microbiota for development and is not freed of this constraint in spite of inhabiting the nutrient-rich environment of *S. purpurea* pitchers.. That (*i*) *W. smithii* first instars hatched from surface-sterilized eggs contain no bacteria or fungi, as demonstrated by our inability to generate viable cultures from pooled homogenates of axenic larvae or PCR amplicons using DNA template from axenic larvae and universal bacterial and fungal primers, (*ii*) almost all of the ASVs we identified in our surface-sterilized larval samples were present in the water from which larvae were collected from, and (*iii*) several of the abundant community members we identified via high-throughput sequencing were able to individually colonize the larval gut also strongly suggests that most of the bacteria we identified in whole-body *W. smithii* larvae and adults via sequencing were present in the digestive tract.

Previous studies in the autogenous mosquito *Aedes atropalpus* have reported similar variability in the developmental outcomes of gnotobiotic larvae colonized by different bacterial isolates [19]. In contrast, the anautogenous mosquito *A. aegypti* develops robustly under variable monocolonized bacterial backgrounds and the same diet conditions [19]. The results herein therefore provide additional evidence to support the hypothesis that fitness of autogenous mosquitoes like *A. atropalpus* and northern populations of *W. smithii* depends on the composition of the gut microbiota and the presence of certain community members. In contrast, the added nutrients obtained through blood feeding obviate such a dependence in anautogenous species like *A. aegypti*. Nevertheless, future work is warranted to examine the robustness of the patterns observed herein, especially given more recent studies underscoring variable impacts of microbiota on mosquito fitness as a function of diet and other rearing factors in the laboratory [84]. Future studies to understand why (and how) certain bacteria enhance or reduce *W. smithii* fitness are also warranted. Recent studies in *A. aegypti* indicate that living microbes induce gut hypoxia and downstream activation of growth-associated signaling pathways by provisioning larvae with riboflavin and other photosensitive B vitamins [85], although the efficiency of riboflavin-based hypoxia induction may vary across different microbial taxa and assemblages as a function of metabolic rate and/or synthetic capacity [75]. While we did not measure the growth rates and associated metabolite profiles of any of the bacterial isolates we assayed, the methodology developed herein strongly positions us to address such questions in the future.

## Conclusions

In this study, we used high throughput 16S rRNA gene amplicon sequencing to characterize microbiota diversity in field and laboratory populations of the pitcher plant mosquito, *W. smithii*, for the first time. We then conducted controlled experiments in the laboratory to better understand the factors shaping microbiota acquisition, persistence, and function in *W. smithii* mosquitoes. Our results support a dominant role for the pitcher environment in shaping microbiota diversity in *W. smithii* larvae, while also indicating that pitcher-associated microbiota can persist in and be dispersed by adult *W. smithii* mosquitoes. We also demonstrate the successful generation of axenic mosquitoes that can be selectively recolonized with one or more known bacterial species in order to study microbiota function. This approach lays the foundation for future work to understand how microbes shape the structure and stability of this model microsystem, with translational significance to other ecosystems of ecological, public health, and agricultural concern. That the axenic larvae generated in this study also failed to grow or molt under normal rearing conditions also contributes to a growing body of literature pertaining to microbial impacts on the physiology and evolution of mosquitoes.

## Supplementary Information

**Additional file 1: Table S 1.** Sequencing and diversity statistics for 16S rRNA gene amplicon libraries prepared from *W. smithii* larvae and water collected from naturally occurring pitchers in the field. Asterisks (*) indicate samples that were removed from the dataset prior to all downstream analyses.

**Additional file 2: Table S 2.** Sequencing and diversity statistics for 16S rRNA gene amplicon libraries prepared from *W. smithii* egg, water, larval, and adult samples collected from our standard laboratory colony. Asterisks (*) indicate samples that were removed from the dataset prior to all downstream analyses.

**Additional file 3: Fig. S 1.** Rarefaction data from Illumina sequences of 16S rRNA gene amplicon libraries prepared from *W. smithii* larvae and water collected from naturally occurring pitchers in the field (*left*) or our standard laboratory colony (*right*).

**Additional file 4: Fig. S 2.** Overlap between ASVs in *W. smithii* larvae and water collected from naturally occurring pitchers in the field and our standard laboratory colony. Values in parentheses indicate the percentage of total ASVs represented in a given sample type or combination of sample types.

**Additional file 5: Fig. S 3.** Rarefaction data from Illumina sequences of 16S rRNA gene amplicon libraries prepared from *W. smithii* egg, water, larval, and adult samples collected from our standard laboratory colony.

**Additional file 6: Fig. S 4.** PCR analysis of *W. smithii* larvae under axenic, gnotobiotic, or conventional conditions. Axenic first instars from surface-sterilized eggs were hatched in closed containers containing sterile water and sterilized diet. Gnotobiotic larvae recolonized by their native microbiota were produced by feeding axenic larvae sterilized diet plus material from a glycerol stock containing the mixed community of bacteria present under conventional rearing conditions. Conventional first instars were hatched in open containers containing distilled water and standard diet. For each treatment, DNA was isolated from a pooled sample of at least 10 larvae after surface sterilization as described herein (see ‘Methods’). DNA samples were then used as template with universal bacterial 16S rRNA gene or fungal ITS primers. The agarose gel shows ethidium bromide-stained PCR products. Lane 1, molecular mass markers labeled in base pairs (bp); Lanes 2–4, universal 16S rRNA gene primers plus DNA from axenic larvae; Lanes 5–7, universal ITS primers plus DNA from axenic larvae; Lanes 8–10, universal 16S rRNA gene primers plus DNA from gnotobiotic larvae; Lanes 11–13, universal 16S rRNA gene primers plus DNA from conventional larvae.

**Additional file 7: Fig. S 5.** Body size (estimated by forewing length) of adult males (*left*) and females (*right*) emerging from experimental plates containing gnotobiotic larvae recolonized by their native microbiota. Adults emerging from trays containing larvae reared conventionally in our standard laboratory colony served as the positive control. A minimum of 600 larvae were assayed per treatment. Box-and-whisker plots show high, low, and median values, with lower and upper edges of each box denoting first and third quartiles, respectively. Plots of the same color represent results from replicate plates (or trays) using larvae derived from independent cohorts of eggs. No significant differences between replicates were detected for adult females (NS). Size likewise did not differ between treatments for adult females (NS; Mann–Whitney U test, *p* > 0.05) after pooling replicates, while gnotobiotic adult males were marginally smaller than conventional males even after accounting for variation between replicates (*; Mann–Whitney U test, *p* < 0.05).

## Data Availability

Raw Illumina reads are available in the NCBI Sequence Read Archive (http://www.ncbi.nlm.nih.gov/sra) under BioProject ID PRJNA767848. Input files for the QIIME pipeline as well as raw data files and R code for statistical analyses have been deposited in the Dryad Digital Repository: https://doi.org/10.5061/dryad.w0vt4b8sg.
