# Supplementary figures and images for: Bacterial communities in carnivorous pitcher plants colonize and persist in inquiline mosquitoes
Source: Anim Microbiome. 2022 Feb 16;4:13. doi: 10.1186/s42523-022-00164-1 (PMC8848819; doi:10.1186/s42523-022-00164-1)

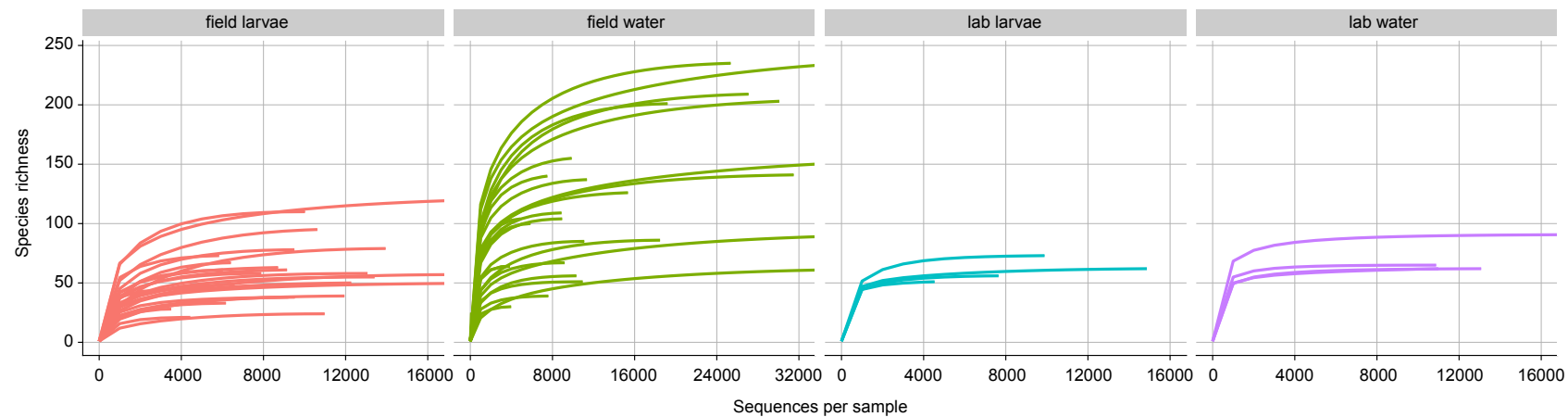

Supplement: Supplementary file 3 — Additional file 3: Fig. S 1. Rarefaction data from Illumina sequences of 16S rRNA gene amplicon libraries prepared from W. smithii larvae and water collected from naturally occurring pitchers in the field (left) or our standard laboratory colony (right). [file 42523_2022_164_MOESM3_ESM.pdf]

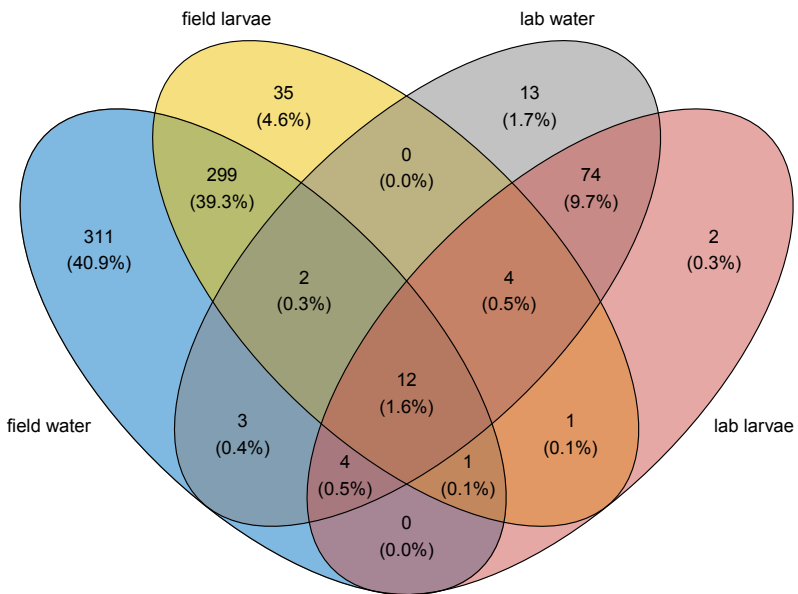

Supplement: Supplementary file 4 — Additional file 4: Fig. S 2. Overlap between ASVs in W. smithii larvae and water collected from naturally occurring pitchers in the field and our standard laboratory colony. Values in parentheses indicate the percentage of total ASVs represented in a given sample type or combination of sample types. [file 42523_2022_164_MOESM4_ESM.pdf]

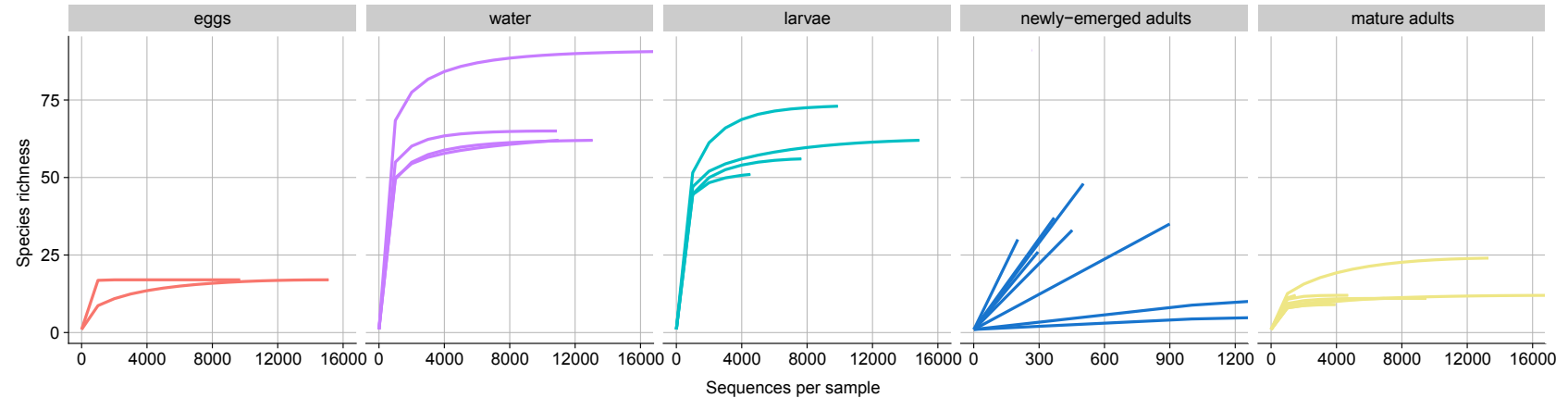

Supplement: Supplementary file 5 — Additional file 5: Fig. S 3. Rarefaction data from Illumina sequences of 16S rRNA gene amplicon libraries prepared from W. smithii egg, water, larval, and adult samples collected from our standard laboratory colony. [file 42523_2022_164_MOESM5_ESM.pdf]

Axenic

Gnotobiotic

Conventional

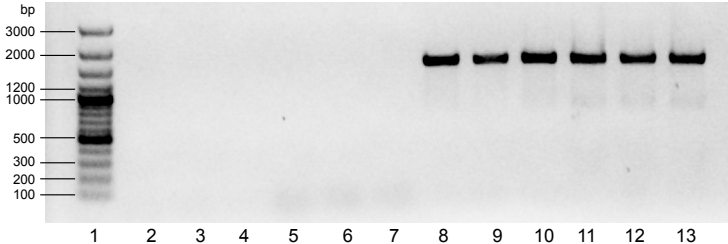

Supplement: Supplementary file 6 — Additional file 6: Fig. S 4. PCR analysis of W. smithii larvae under axenic, gnotobiotic, or conventional conditions. Axenic first instars from surface-sterilized eggs were hatched in closed containers containing sterile water and sterilized diet. Gnotobiotic larvae recolonized by their native microbiota were produced by feeding axenic larvae sterilized diet plus material from a glycerol stock containing the mixed community of bacteria present under conventional rearing conditions. Conventional first instars were hatched in open containers containing distilled water and standard diet. For each treatment, DNA was isolated from a pooled sample of at least 10 larvae after surface sterilization as described herein (see ‘Methods’). DNA samples were then used as template with universal bacterial 16S rRNA gene or fungal ITS primers. The agarose gel shows ethidium bromide-stained PCR products. Lane 1, molecular mass markers labeled in base pairs (bp); Lanes 2–4, universal 16S rRNA gene primers plus DNA from axenic larvae; Lanes 5–7, universal ITS primers plus DNA from axenic larvae; Lanes 8–10, universal 16S rRNA gene primers plus DNA from gnotobiotic larvae; Lanes 11–13, universal 16S rRNA gene primers plus DNA from conventional larvae. [file 42523_2022_164_MOESM6_ESM.pdf]

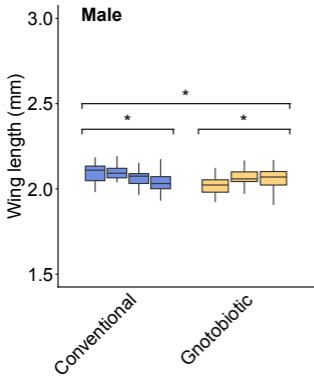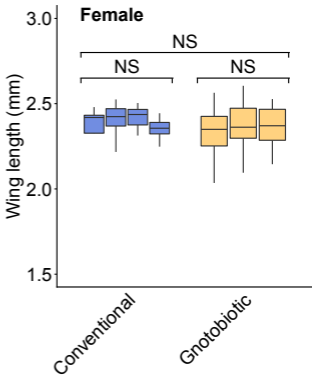

Supplement: Supplementary file 7 — Additional file 7: Fig. S 5. Body size (estimated by forewing length) of adult males (left) and females (right) emerging from experimental plates containing gnotobiotic larvae recolonized by their native microbiota. Adults emerging from trays containing larvae reared conventionally in our standard laboratory colony served as the positive control. A minimum of 600 larvae were assayed per treatment. Box-and-whisker plots show high, low, and median values, with lower and upper edges of each box denoting first and third quartiles, respectively. Plots of the same color represent results from replicate plates (or trays) using larvae derived from independent cohorts of eggs. No significant differences between replicates were detected for adult females (NS). Size likewise did not differ between treatments for adult females (NS; Mann–Whitney U test, p > 0.05) after pooling replicates, while gnotobiotic adult males were marginally smaller than conventional males even after accounting for variation between replicates (*; Mann–Whitney U test, p < 0.05). [file 42523_2022_164_MOESM7_ESM.pdf]
